# Breastfeeding-related neck pain: prevalence and correlates among Nigerian lactating mothers

**DOI:** 10.1093/inthealth/ihac050

**Published:** 2022-07-23

**Authors:** Chidiebele Petronilla Ojukwu, Chinechendu Glory Okpoko, Adaora Justina Okemuo, Stephen Sunday Ede, Ijeoma Judith Ilo

**Affiliations:** Department of Medical Rehabilitation, College of Medicine, University of Nigeria Enugu, Nigeria; Department of Medical Rehabilitation, College of Medicine, University of Nigeria Enugu, Nigeria; Department of Medical Rehabilitation, College of Medicine, University of Nigeria Enugu, Nigeria; Department of Medical Rehabilitation, College of Medicine, University of Nigeria Enugu, Nigeria; Department of Physiotherapy, College of Allied Health Sciences, Gregory University, Uturu, Nigeria; Department of Nursing Sciences, College of Medicine, University of Nigeria Enugu, Nigeria

**Keywords:** breastfeeding characteristics, lactating mothers, neck pain, postpartum

## Abstract

**Background:**

Breastfeeding (BF) is a physically demanding task and is predominantly performed in a head-down position as the mother attempts to maintain eye contact with the infant. There are possibilities of BF-related neck pain (BFRNP) that have not been widely studied. This study investigated the prevalence and correlates of BFRNP in Nigerian lactating mothers.

**Methods:**

A cross-sectional descriptive survey was conducted among 310 lactating mothers selected from post-natal clinics in Enugu, Nigeria. Information on their BF profile was sought as well as the prevalence and characteristics of BFRNP using a self-structured questionnaire.

**Results:**

It was found that cradle hold was the commonly adopted BF position (94.0%) and the majority breastfed ≥10 times daily (55.2%) for <30 min per session (49.1%). BFRNP was seen in 51.7% of women, of which 55.0% reported moderate pain intensity and 60.0% experienced this pain during BF. None of the maternal characteristics and breastfeeding profiles were significantly associated with the prevalence of BFRNP (p>0.05).

**Conclusions:**

There is a high prevalence of BFRNP among nursing mothers. Although maternal characteristics and BF positions were not associated with reported BFRNP, the results suggest that changing BF positions reduces neck pain during nursing sessions. Therefore this study recommends that nursing mothers should regularly change their BF positions to increase relaxation and comfort.

## Introduction

Breastfeeding (BF) is one of the most effective ways to ensure child health and survival.^1^ Recently there has been an increased campaign to reduce barriers to the early initiation of BF and encourage exclusive BF as recommended by the United Nations Children's Fund (UNICEF) and World Health Organization (WHO).^2,3^ Such campaigns have been geared towards maximizing the benefits of BF for the mother and infant.^1,4^ Despite the common benefits of BF, it is not without a cost, as it weighs heavily in the life-altering and immense responsibility of having a new baby.^5^ Musculoskeletal pains^6–8^ are among the common challenges experienced by BF mothers, in addition to breast soreness, engorgement, maternal illness, blocked ducts and mastitis.^9^

From previous studies,^10–12^ BF-related musculoskeletal problems have been commonly associated with the adoption of awkward postures during feeding sessions. From anecdotal observations, one of the most common awkward positions adopted by nursing mothers is unsupported head/neck posture with resultant sustained neck flexion in an attempt to monitor the infant during feeding. Such a sustained awkward position with excessive repetition usually puts stress on the neck and back muscles.^13^ BF-related neck pain (BFRNP) has been reported among nursing mothers, particularly those who adopt unsuitable BF postures. Mbada et al.^8^ reported a high prevalence of BF-related musculoskeletal pain, including neck pain, among Nigerian nursing mothers. Similarly, Rani et al.^12^ reported mechanical neck pains among lactating mothers in Rawalpindi and Islamabad. In these studies, the BF position, which is an important characteristic of BF, was assessed and related with the reported prevalence. Mbada et al.^8^ showed that BFRNP was more prevalent among women who breastfed while sitting on a mat or bedside. Rani et al.^12^ also observed a higher prevalence of musculoskeletal pain among women who used the cross-cradle hold, although there was no statistical significance in this association. However, these studies were not exhaustive in the assessment of BF-related musculoskeletal pain relative to a wider range of BF characteristics.

Factors characterizing BF practices may vary between or within nursing mothers. Such characteristics may include the adopted BF position(s), duration and frequency of BF, practice of exclusive BF and number of breastfed children. It is necessary to ascertain the relative associations of these characteristics with the prevalence of BFRNP to guide maternal education on the prevention of BFRNP. Therefore this study was designed to assess the prevalence and correlates of BFRNP among Nigerian nursing mothers.

## Methods

### Design

This is a cross-sectional descriptive survey investigating the prevalence and association of BF characteristics with BFRNP in lactating mothers.

### Participants

The participants in this study were all nursing mothers who were 18–40 y of age and currently BF their infants. Women who were pregnant and nursing with known orthopaedic and neurological conditions of the spine, upper limbs and shoulder regions prior to BF were excluded from this study. A priori power analysis to determine the sample size needed to achieve 90% power with a moderate effect size of 0.50 at a level of significance supported a total sample of 310 women for this study.^14^

### Protocol

Ethical approval for this study was first obtained from the University of Nigeria Research Ethics Committee, Enugu State of Nigeria before commencement of the study. Respondents were conveniently recruited from five postnatal clinics in Enugu State, Nigeria. Postpartum women who attended these clinics on the study days were collectively addressed on the details of the study. Volunteers who met the study eligibility criteria signed informed consent forms prior to participation in the survey. Subsequently the study questionnaire was administered to each respondent on a face-to-face basis and collected immediately after they responded.

### Questionnaire

This was a structured questionnaire with three sections (A, B and C). Section A elicited data on the sociodemographic and maternal characteristics of the respondent, which included the participant's age, religion, occupation, marital status, number of breastfed children and postpartum duration, among others. Section B sought information on the BF characteristics of the respondents, including the duration of BF, practice of exclusive BF, frequency of BF and commonly adopted BF positions with pictorial representations of the three common BF positions (cradle hold, cross-cradle hold and the clutch or football hold) adopted by Nigerian nursing mothers.^15^ Section C sought information on the prevalence and characteristics of BFRNP.

This questionnaire was face and content validated by four experts. Its reliability was assessed in a pilot study by a test–retest method among 10 nursing mothers from one of the postnatal clinics. A total of 10 d elapsed between the test and retest activities. The correlation coefficient of the test–retest reliability was r=0.977 (p=0.001).

A total of 480 copies of the questionnaire were printed and distributed to the nursing mothers at the postnatal clinics and 310 mothers completed questionnaires were collected for data collation and analysis, yielding a response rate of 64.6%.

## Data analysis

Descriptive statistics of frequencies and percentages were used to summarize the responses. Inferential statistics of χ^2^ were used to evaluate associations among the prevalence of BFRNP, maternal characteristics and the BF profile of the respondents. Data were analysed with SPSS version 20.0 (IBM, Armonk, NY, USA).

## Results

The sociodemographic, maternal characteristics and BF factors affecting BFRNP are shown in Table 1. We found that the majority of mothers were 26–30 y of age (63.7%), married (94%), Christian (96.6%) and self-employed (62.9%).

**Table 1.** Factors (maternal characteristics and breastfeeding profile) affecting BFRNP (N=310)

Variables	n (%)	BFRNP (n=160), n (%)	p-Value
Age (years)			
18–25	93.2 (30.2)	51 (31.7)	0.542
26–30	134 (43.1)	67 (41.7)	
31–35	56 (18.1)	24 (15.0)	
36 and above	27 (8.6)	18 (11.6)	
Marital status			
Single	19 (6.0)	11 (6.7)	0.767
Married	291 (94.0)	149 (93.3)	
Religion			
Christian	299 (96.5)	152 (95.0)	0.272
Islam	5 (1.7)	5 (3.3)	
Traditional religion	3 (0.9)	0 (0)	
Others	3 (0.9)	3 (1.7)	
Occupation			
Self-employed	230 (74.1)	139 (86.7)	0.241
Civil servant	51 (16.4)	19 (11.7)	
Unemployed	29 (9.5)	2 (1.6)	
Number of children			
1	109 (35.3)	53 (33.3)	0.970
2–4	171 (55.2)	91 (56.7)	
≥5	30 (9.5)	16 (10.0)	
Number of breastfed children			
1	118 (37.9)	59 (36.6)	0.353
2–4	166 (53.4)	88 (55.0)	
≥5	26 (8.6)	13 (8.4)	
Postpartum duration (months)			
1–3	166 (53.4)	83 (51.7)	0.468
4–6	72 (23.3)	32 (20.0)	
7–9	43 (13.8)	29 (18.3)	
≥10	29 (9.5)	16 (10.0)	
Baby weight (kg)			
<5	83 (26.7)	43 (26.7)	0.809
5–10	181 (58.6)	107 (66.7)	
>10	46 (14.7)	10 (6.6)	
Currently breastfeeding			
Yes	310 (100)	160 (100)	1.000
No	0 (0)	0 (0)	
Exclusive BF			
Yes	152 (49.1)	88 (55.0)	0.131
No	158 (50.9)	72 (45.0)	
Duration of BF (months)			
1–3	166 (53.4)	83 (51.7)	0.468
4–6	72 (23.3)	32 (20.0)	
7–9	43 (13.8)	29 (18.3)	
≥10	29 (9.5)	16 (10.0)	
Frequency of BF (sessions per day)			
1–3	40 (12.9)	20 (12.5)	0.970
4–6	24 (7.8)	18 (11.5)	
7–9	72 (23.3)	33 (20.6)	
≥10	174 (56.0)	89 (55.6)	
Duration of BF (minutes per session)			
<30	152 (49.1)	80 (50.0)	0.630
30–60	128 (41.4)	61 (38.3)	
>60	30 (8.6)	19 (11.7)	
Commonly adopted BF positions			
Cradle hold	291 (94.0)	149 (93.3)	0.577
Cross-cradle hold	13 (4.3)	8 (5.0)	
Football hold	6 (0.9)	2 (1.7)	

The general maternal characteristics and BF profile of the respondents are shown in Table 2. The majority had undergone two to four childbirths (55.2%), had breastfed two to four children (53.4%) and were within 1–3 months after childbirth during the data collection (53.4%). Regarding their BF profiles, the majority had been breastfeeding for 1–3 months (53.4%), were not BF exclusively (50.9%), breastfed >10 times a day (55.2%) for <30 min per session (49.1%) and adopted the cradle hold BF position (94%).

**Table 2.** Prevalence and characteristics of BFRNP among the respondents

Variables	Frequency	Percent
Neck pain		
Yes	160	51.7
No	150	48.3
Total	310	100
Pain characteristics (n=160)		
Severity of neck pain		
Mild	61	38.3
Moderate	88	55.0
Severe	11	6.7
Time of occurrence		
During breastfeeding	96	60
After breastfeeding	37	23.3
During and after breastfeeding	27	16.7
Frequency of occurrence of neck pain		
Every time	27	16.7
Sometimes	109	68.3
Once in a while	24	15
Duration of neck pain (minutes per pain experience)		
<10	85	53.3
10–20	64	40
≤20	11	6.7
BF position that causes pain		
Cradle hold	147	91.7
Cross-cradle	13	8.3
Football	0	0
Pain reduction with change of BF posture		
Yes	133	83.3
No	27	16.7
Previous experience of BF-related neck pain		
Yes	59	37.1
No	101	62.9

Table 1 also presents the χ^2^ test results showing associations among prevalence of BFRNP, maternal characteristics and the BF profile of the women. The results revealed that none of these factors was significantly (p>0.05) associated with the prevalence of BFRNP in these nursing mothers.

The prevalence and characteristics of BFRNP among nursing mothers is shown in Table 2. The results showed that 51.7% of the respondents experienced BFRNP. For the majority, the experienced neck pain was moderate (55%), occurred sometimes (68.3%), occurred during BF (60%) and usually lasted for <10 min (53.3%). The cradle hold BF position elicited the highest pain in most (91.7%) of the participants as compared with other BF positions. The majority (83.3%) of the women also reported that changing BF positions reduced their neck pain during nursing sessions.

## Discussion

### Prevalence of BFRNP among Nigerian lactating mothers

Visualizing the act of BF suggests the possibility of associated musculoskeletal discomfort in nursing mothers. This study revealed that the prevalence of BFRNP is high among Nigerian nursing mothers. Neck pain has been identified as a common musculoskeletal disorder in nursing mothers.^6,8,12,16,17^ BFRNP has been attributed to several factors. Kyphotic posture during BF may result to increased intradisk pressure and potential microtrauma to the spinal tissues.^18^ Such kyphotic postures usually result from physiologic changes that alter the centre of gravity, resulting in tight neck and shoulder muscles in combination with overstretched abdominals and paraspinal muscles.^17^ The persistence of pregnancy-related hormonal influences on ligaments and connective tissues may result to joint hypermobility and increased risk of injuries in postpartum women.^19,20^ Lactation-related hormonal influences may also result in musculoskeletal pain.^6^ Posture is one of the major factors that influences the incidence of musculoskeletal pain in nursing mothers. Body posture and positioning are integral parts of BF, but adoption of the wrong posture will likely result in different musculoskeletal disorders, including BFRNP.^8,12^

BF can be categorized as a prolonged task, considering the duration and frequency involved, particularly in the case of exclusive BF. According to Mbada et al.,^8^ inappropriate posture while performing a task for a long period can lead to end-range loading of muscles and deformation of normal body tissue, thus leading to musculoskeletal disorders, including neck pain in the case of BF. In addition, the act of a mother trying to maintain her gaze on the nursing infant results in prolonged neck flexion with increased cervical kyphosis. The subsequent musculoskeletal strain while adopting this posture is a predisposing factor for BFRNP.^8,12^ For ease and improved latching, mothers have been observed to lean forward towards the baby during BF, particularly when nursing small or younger babies. Such a posture produces protracted shoulders, increased strain on the posterior neck and upper back muscles, as well as shortened anterior neck and shoulder muscles.^21^ Thus nursing mothers are discouraged from leaning forward to bring the breast to the baby; rather the baby should be brought to the breast.^17^

Characteristically, the BFRNP reported by nursing mothers in the present study was predominantly of moderate intensity and mostly occurred during the BF task rather than before and after feeding sessions. Logically, the mechanisms responsible for neck pain are usually triggered during BF. However, some women also reported persistence of symptoms after BF. Most women associated their experience of BFRNP with the cradle hold BF position as compared with the cross-cradle and football holds. This is contrary to the findings of Rani et al.,^12^ who reported that nursing mothers in Pakistan associated the cross-cradle hold with a higher prevalence of BFRNP. In a recent study, Ojukwu et al.^22^ assessed the electromyographic activities of the neck muscles during different BF holds. Their findings showed that neck muscle activities were lower during the cradle hold position as compared with the cross-cradle and football holds. They further recommended that the cradle hold is the most biomechanically efficient BF hold position. The contradictory report from the present study may result from the fact that most of the women commonly adopted the cradle hold position, resulting in a numerical bias suggesting this position as a common factor of BFRNP. The majority of the mothers also reported that changing BF positions reduced their BFRNP. Previous studies^15,22^ recommended alternating BF positions within and between BF sessions as a strategy for preventing or reducing BF-related musculoskeletal disorders.

Furthermore, this study showed no association among the prevalence of BFRNP, maternal factors and the BF characteristics of the mothers. However, marginal differences in prevalence rates relative to these factors and characteristics were observed. For instance, multiparous women who had nursed two to four children reported more pain than those with fewer or more than five children. Hogg-Johnson et al.^23^ opined that larger numbers of breastfed children resulted in more and worsening BF-related pains among nursing mothers. Mothers in the present study also experienced more pain within the postpartum duration of 1–3 months. This period corresponds to the post-pregnancy recovery phase, characterized by reduced musculoskeletal integrity and a higher risk of pain.^6,20^ According to Stainton et al.,^24^ early postpartum experiences are characterized by more pain, which declines progressively as the mother recuperates.

The BF characteristics with a higher prevalence of BFRNP include the practice of exclusive BF, BF frequency >10 times per day and adoption of the cradle hold BF position. Exclusive BF implies higher frequencies of BF and would be expected to result in higher incidences of BFRNP. Increasing frequencies of BF have also been attributed to higher risks of BFRNP.^23^ This explains the higher prevalence of BFRNP in mothers who breastfed >10 times per day compared with those with lower daily BF frequencies. Similar to the present study's findings, Rani et al.^12^ also showed no significant differences between BFRNP and BF positions utilized by Pakistani mothers.

Despite the absence of statistically significant associations among BF prevalence, maternal factors and BF characteristics, the variations in the distribution of BFRNP relative to these characteristics suggest the need for further studies to explore possible associations. The high prevalence of BFRNP also creates the need for intensified maternal education on BF ergonomics. Thus women's health physiotherapists, occupational therapists and lactation consultants need to engage in maternal health promotion programs to curb the prevalence of BFRNP as well as improve BF outcomes in mothers and infants. A potential limitation of this study is the subjectivity in pain assessments, which was basically based on self-reports. Inappropriate recall of BF experiences may have also affected the responses of the mothers.

## Conclusions

There is a high prevalence of BFRNP among nursing mothers. Although maternal characteristics and BF positions were not associated with reported BFRNP, our results suggest that changing BF positions reduces neck pain during nursing sessions. Therefore this study recommends that nursing mothers should regularly change their BF positions to increase relaxation and comfort.

## Data Availability

Due to privacy and ethical concerns, data for this study will be made available on request.
